# Prognostic value of preoperative combined neutrophil, monocyte, and lymphocyte scores in patients with renal cell carcinoma undergoing laparoscopic nephrectomy: A retrospective study

**DOI:** 10.1002/cam4.7214

**Published:** 2024-04-30

**Authors:** Jinliang Ni, Xiaoxiang Yao, Wei Song, Heng Zhang, Houliang Zhang, Yidi Wang, Yifan Zhang, Guangchun Wang, Keyi Wang, Weipu Mao, Bo Peng

**Affiliations:** ^1^ Department of Urology, Shanghai Putuo District People's Hospital, School of Medicine Tongji University Shanghai China; ^2^ Department of Urology, Shanghai Tenth People's Hospital, School of Medicine Tongji University Shanghai Shanghai China; ^3^ Department of Oncology, Putuo People's Hospital, School of Medicine Tongji University Shanghai China; ^4^ Shanghai Clinical College Anhui Medical University Shanghai China; ^5^ Department of Urology Guiqian International General Hospital Guizhou China; ^6^ Department of Urology Affiliated Zhongda Hospital of Southeast University Nanjing China; ^7^ Department of Urology The First Affiliated Hospital of Zhengzhou University Zhengzhou China

**Keywords:** immune cells, laparoscopic nephrectomy, peripheral blood immune score, prognosis, renal cell carcinoma

## Abstract

**Background:**

In a multi‐institutional clinical study, we assessed the prognostic significance of a novel indicator preoperative peripheral blood immune (PBIS) scores that combined ratios of preoperative lymphocyte, monocyte, and neutrophil of renal cell carcinoma (RCC) patients undergoing laparoscopic nephrectomy.

**Methods:**

Between January 2014 and December 2019, 438 patients with RCC were retrospectively analyzed in three centers. We used X‐tile software to obtain the optimum cut‐off values for neutrophils, monocytes, and lymphocytes to classify the patients. To assess the relationship between PBIS score and overall survival (OS), and cancer‐specific survival (CSS) in patients with RCC by Kaplan–Meier survival curves and Cox regression analyses. In addition, predictive OS and CSS nomograms were constructed. The discriminative ability of nomogram and predictive performance accuracy were verified with consistency index (C‐index), calibration curves, receiver operating curve (ROC) curves, decision curve analysis (DCA) curves, and time‐dependent ROC curves.

**Results:**

The optimum cutoff values for monocytes, lymphocytes, and neutrophils were 0.46, 1.01, and 4.50, respectively. We divided patients into four subgroups according to PBIS scores, which were significantly associated with M‐stage (*p* = 0.008), T‐stage (*p* < 0.001), N‐stage (*p* = 0.006), and AJCC stage (*p* < 0.001). Multivariate Cox regression analysis revealed that RCC patients with lower PBIS scores showed a worse postoperative prognosis and served as an independent predictor of OS (*p* = 0.002) and CSS (*p* < 0.001). Ultimately, the nomograms based on PBIS scores demonstrated excellent predictive performance for OS (C‐index: 0.770) and CSS (C‐index: 0.828) through the analysis of calibration curves, ROC curves, DCA curves, and time‐dependent ROC curves.

**Conclusion:**

PBIS score served as novel and effective predictor to accurately predict OS and CSS in patients with RCC receiving laparoscopic nephrectomy.

## 1 INTRODUCTION

In 2022, according to epidemiological studies, renal cancer is considered to be the ninth most common cancer in men and the fourteenth in women.^1^ The standard prognosis for renal cancer is tumor characteristics such as nodal stage, cancer stage, nuclear grading, and metastatic stage.^2^ In addition, there are a variety of models that incorporate patients' clinical characteristics to predict prognosis, including the UCLA Integrated Staging System (UISS), Memorial Sloan‐Kettering Cancer Center (MSKCC/Motzer) Score, and International Metastatic Renal Cell Carcinoma Database Consortium (IMDC) risk model.^3^, ^4^ But with the tumor regression and the switch of cancer stage after chemotherapy and radiotherapy, it is hard to provide complete prognostic information.^5^, ^6^ It obscures the postoperative adjuvant treatment of patients. Most importantly, there are as yet no desirable biomarkers to predict accurately the prognosis of RCC patients after treatment.

The correlation between cancer and inflammation has been elucidated.^7^, ^8^ Anti‐inflammatory drugs such as Polyphenols or Brusatol significantly lowered the risk of kidney cancer.^9^ Previous studies have found significant changes in peripheral blood leukocyte counts in RCC cases and correlated with prognosis.^10^ Furthermore, a consensus immunoscore, a scoring system that summarizes the density of CD8^+^ and CD3^+^ T‐cell effectors in tumors and their infiltrating margins, has been demonstrated to be a reliable estimate of the risk of recurrence risk in colon cancer patients.^11^ Various cancer types were covered by this kind of assessment that the increasing levels of monocytes and neutrophils in peripheral blood, or the decreasing levels of lymphocytes refer to the poor state of prognosis to individuals in corresponding tumors.^12^, ^13^ Therefore, peripheral blood immune cells may be able to comprehensively assess the efficacy of therapeutic strategies in terms of anti‐tumor or pro‐tumor immune responses and may be useful in predicting the prognosis of patients with renal cancer. But the effect or the accuracy of this kind of immunoscore system in kidney cancer is still ready to be proved.

Hence, this study was conducted to systematically assess the association between preoperative peripheral blood immune cells and the prognosis of RCC patients who underwent laparoscopic nephrectomy, including neutrophils, monocytes, and lymphocytes. Meanwhile, we constructed a novel index peripheral blood immune (PBIS) scores constructed nomograms to assess the prognosis of patients with RCC to facilitate optimal clinical decision‐making by urologists.

## 2 PATIENTS AND METHODS

### 2.1 Patients

Altogether, at Zhongda Hospital of Southeast University, Shanghai Shidong Hospital, and the Shanghai Tenth People's Hospital of Tongji University from January 2014 to December 2019, 590 patients with RCC who underwent partial or radical nephrectomy were collected in the present multi‐institutional study. This study was approved and supervised by the Ethics Committees and Institutional Review Boards of all participating institutions (SHSY‐IECKY‐4.0/18‐68/01 and ZDKYSB077) and was conducted in accordance with the standards set out in the Declaration of Helsinki (revised 2013). Each patient or his/her relative who participated in this study signed an informed consent form.

The criteria for inclusion of patients were determined as follows: (1) patients diagnosed with RCC by histopathology; (2) patients treated with laparoscopic nephrectomy; (3) patients with complete medical records and no missing follow‐up data. The exclusion criteria are established as follows: (1) patients with a combination of other malignancies that seriously affect survival; (2) patients received other anti‐cancer treatments prior to laparoscopic nephrectomy; (3) patients with pre‐operative blood or other autoimmune diseases. According to these criteria, we excluded 152 patients, and 438 patients were finally included in this study.

### 2.2 Clinical data collection and follow‐up

We retrospectively retrieved and obtained clinicopathological features, laboratory test data, and baseline data for all patients from the electronic medical records of all participating institutions. Clinicopathological features of RCC patients included TNM stage (8th edition), American Joint Committee on Cancer (AJCC) stage and Fuhrman grade. Data on patients' baseline characteristics were incorporated for gender, age, body mass index [BMI, weight (kg)/height^2^ (m^2^)], history of cardiovascular diseases, history of diabetes, history of hypertension, survival time, and smoking. All laboratory test data were examined within 2 days prior to surgery and further collected, including the counts of neutrophils, lymphocytes, and monocytes. Hematology samples from this study were collected by trained professionals and analyzed for biochemical composition with the same automated system. Outpatient or telephone follow‐up was performed for all patients undergoing nephrectomy and discharged from the hospital. Patients were followed up at 3–4 month intervals for the first 2 years after treatment and at 6 month intervals thereafter until loss to follow‐up or death. The time from the end of surgery to the end of follow‐up or death was defined as patient survival time. Cancer‐specific survival (CSS) was defined as the time between the date of therapeutic resection and the date of death due to RCC. It was defined as the overall survival (OS) from the date of surgical treatment to the date of death or the last follow‐up visit.

### 2.3 Definition of PBIS

The optimal cutoff values for neutrophils, lymphocytes, and monocytes were analyzed using the X‐tile program (version 3.6.1). High and low levels of the three immune cells were classified into groups based on optimal cutoff values. It was defined as a score of 1 given that a high neutrophil count, high monocyte count, and low lymphocyte count were considered unfavorable for the prognosis of patients with RCC. Patients with low neutrophil count, high lymphocyte count, and low monocyte count were scored 0. Finally, according to the total score obtained by summing the three cell scores of the patients, the PBIS scoring system was classified into four groups, namely, PBIS 0 group, PBIS 1 group, PBIS 2 group, and PBIS 3 group.^2^

### 2.4 Statistical analysis

All data analyses in the present study were performed with SPSS software (version 26.0) and RStudio software (version 2023.03.0 + 386). For categorical variables, Fisher's exact test or Chi‐square test were used to analyze the differences between the two groups, and for continuous variables, differences between the two groups were analyzed using Student's *t*‐test. For categorical variables, it is presented as number (%), and for continuous variables, it is presented as mean ± standard deviation (SD). The effects of neutrophils, lymphocytes and monocytes, and PBIS score on survival outcomes were evaluated with Kaplan–Meier curves and tested with log‐rank test. Univariate and multivariate Cox proportional hazards regression models were performed to assess independent risk factors for OS and CSS. The hazard ratios (HRs) calculated from the Cox regression models were taken as relative risks with 95% confidence interval (CI). Predictive nomograms with all independent prognostic factors related to OS and CSS were established using RStudio software. Internal and external verification of the nomograms were evaluated with bootstrap resampling and 10‐fold cross‐validation to plot the calibration curves. Decision curve analysis (DCA), Harrell's concordance index (C index) and receiver operating characteristic (ROC) curves were conducted to assess potential clinical discernment and efficacy of the nomogram. The predictive ability of nomograms, AJCC stage, and PBIS score for OS and CSS was compared with the area under the curve (AUC) of the time‐dependent ROC curves. When *p* < 0.05, statistical results were considered significant.

## 3 RESULTS

### 3.1 Correlation of different immune cells with clinical prognosis and clinicopathological characteristics

The optimal cutoff values for neutrophils, lymphocytes, and monocytes were 4.50, 1.01, and 0.46, respectively (Figure S1). Patients were then categorized into high neutrophil (>4.5; *n* = 159) or low neutrophil (≤4.50; *n* = 279) groups, high monocyte (>0.46; *n* = 110) or low monocyte (≤0.46; *n* = 328) groups and high lymphocyte (>1.01 *n* = 392) or low lymphocyte (≤1.01; *n* = 46) groups according to cut‐off values. The associations of different preoperative immune cells with other clinicopathological characteristics were summarized in Table 1. The median follow‐up time for patients included in this study was 32 months. By the end of follow‐up, 54 patients (12.3%) had died, of whom 36 (8.2%) died of RCC. The results by Chi‐square test indicated that neutrophil level was statistically related to T‐stage (*p* = 0.021), M‐stage (*p* = 0.045), and AJCC stage (*p* = 0.014). Monocyte level was statistically associated with T‐stage (*p* = 0.003) and AJCC stage (*p* = 0.015). And lymphocyte level was statistically correlated with BMI (*p* = 0.011), T‐stage (*p* = 0.004), Fuhrman grade (*p* = 0.047), N‐stage (*p* = 0.009), M‐stage (*p* = 0.022), and AJCC stage (*p* < 0.001).

**TABLE 1:** Association between immune cells (neutrophils, lymphocytes and monocytes) and clinicopathological characteristics.

Characteristics	All patients	Neutrophil			Monocyte			Lymphocyte		
		≤4.50	>4.50	*p‐*value	≤0.46	>0.46	*p‐*value	≤1.01	>1.01	*p*‐value
		*N* = 279	*N* = 159		*N* = 328	*N* = 110		*N* = 46	*N* = 392	
Age, years										
≤65	316 (72.1)	200 (71.7)	116 (73.0)	0.775	239 (72.9)	77 (70.0)	0.562	29 (63.0)	287 (73.2)	0.145
>65	122 (27.9)	79 (28.3)	43 (27.0)		89 (27.1)	33 (30.0)		17 (37.0)	105 (26.8)	
Sex										
Male	293 (66.9)	183 (65.6)	110 (69.2)	0.443	213 (64.9)	80 (72.7)	0.133	24 (52.2)	269 (68.6)	0.025
Female	145 (33.1)	96 (34.3)	49 (30.8)		115 (35.1)	30 (27.3)		22 (47.8)	123 (31.4)	
BMI, kg/m^2^										
≤24	247 (56.4)	160 (57.3)	87 (54.7)	0.593	186 (56.7)	61 (55.5)	0.819	34 (73.9)	213 (54.3)	0.011
>24	191 (43.6)	119 (42.7)	72 (45.3)		142 (43.3)	49 (44.5)		12 (26.1)	179 (45.7)	
Hypertension										
No	250 (57.1)	156 (55.9)	94 (59.1)	0.515	195 (59.5)	55 (50.0)	0.083	32 (69.6)	218 (55.6)	0.070
Yes	188 (42.9)	123 (44.1)	65 (40.9)		133 (40.5)	55 (50.0)		14 (30.4)	174 (44.4)	
Diabetes										
No	368 (84.0)	231 (82.8)	137 (86.2)	0.355	276 (84.1)	92 (83.6)	0.899	38 (82.6)	330 (84.2)	0.783
Yes	70 (16.0)	48 (17.2)	22 (13.8)		52 (15.9)	18 (16.4)		8 (17.4)	62 (15.8)	
Cardiovascular diseases										
No	387 (88.4)	244 (87.5)	143 (89.9)	0.436	290 (88.4)	97 (88.2)	0.947	39 (84.8)	348 (88.8)	0.424
Yes	51 (11.6)	35 (12.5)	16 (10.1)		38 (11.6)	13 (11.8)		7 (15.2)	44 (11.2)	
Smoking										
No	365 (83.3)	230 (82.4)	135 (84.9)	0.505	277 (84.5)	88 (80.0)	0.278	39 (84.8)	326 (83.2)	0.780
Yes	73 (16.7)	49 (17.6)	24 (15.1)		51 (15.5)	22 (20.0)		7 (15.2)	66 (16.8)	
T‐stage										
T1	333 (76.0)	223 (79.9)	110 (69.2)	0.021	261 (79.6)	72 (65.5)	0.003	27 (58.7)	306 (78.1)	0.004
T2	30 (6.8)	20 (7.2)	10 (6.3)		22 (6.7)	8 (7.3)	0.108	6 (13.0)	24 (6.1)	
T3	64 (14.6)	31 (11.1)	33 (20.8)		36 (11.0)	28 (25.5)		9 (19.6)	55 (14.0)	
T4	11 (2.5)	5 (1.8)	6 (3.8)		9 (2.7)	2 (1.8)		4 (8.7)	7 (1.8)	
N‐stage										
N0	421 (96.1)	270 (96.8)	151 (95.0)	0.347	315 (96.0)	106 (96.4)	0.878	41 (89.1)	380 (96.9)	0.009
N1	17 (3.9)	9 (3.2)	8 (5.0)		13 (4.0)	4 (3.6)		5 (10.9)	12 (3.1)	
M‐stage										
M0	419 (95.7)	271 (97.1)	148 (93.1)	0.045	316 (96.3)	103 (93.6)	0.228	41 (89.1)	378 (96.4)	0.022
M1	19 (4.3)	8 (2.9)	11 (6.9)		12 (3.7)	7 (6.4)		5 (10.9)	14 (3.6)	
Fuhrman grade										
I	73 (16.7)	55 (19.7)	18 (11.3)	0.068	53 (16.2)	20 (18.2)	0.651	3 (6.5)	70 (17.9)	0.047
II	273 (62.3)	173 (62.0)	100 (62.9)		208 (63.4)	65 (59.1)		29 (63.0)	244 (62.2)	
III	82 (18.7)	45 (16.1)	37 (23.3)		61 (18.6)	21 (19.1)		11 (23.9)	71 (18.1)	
IV	10 (2.3)	6 (2.2)	4 (2.5)		6 (1.8)	4 (3.6)		3 (6.5)	7 (1.8)	
AJCC stage										
I	326 (74.4)	217 (77.8)	109 (68.6)	0.014	256 (78.0)	70 (63.6)	0.015	26 (56.5)	300 (76.5)	<0.001
II	26 (5.9)	19 (6.8)	7 (4.4)		19 (5.8)	7 (6.4)		5 (10.9)	21 (5.4)	
III	61 (13.9)	33 (11.8)	28 (17.6)		37 (11.3)	24 (21.8)		6 (13.0)	55 (14.0)	
IV	25 (5.7)	10 (3.6)	15 (9.4)		16 (4.9)	9 (8.2)		9 (19.6)	16 (4.1)	

*Note*: Continuous data are presented as the mean ± standard deviation and categorical data as *n* (%). For categorical variables, *p*‐values were analyzed by chi‐square tests. For continuous variables, the *t*‐test for slope was used in generalized linear models.

Abbreviations: AJCC, American Joint Committee on Cancer; BMI, body mass index; PBIS score, peripheral blood immune score.

### 3.2 Prognostic effects of preoperative immune cells and PBIS scores on RCC patients

**FIGURE 1:**
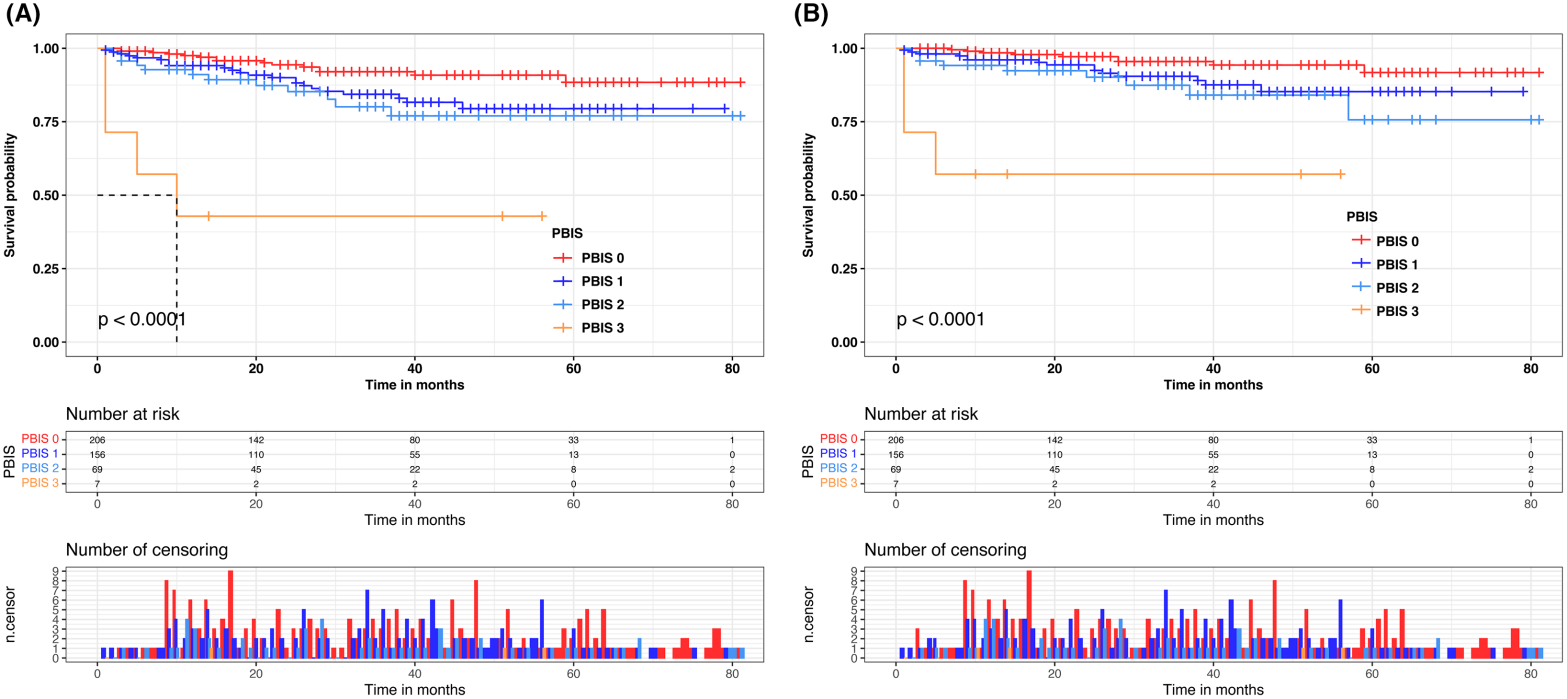
Overall survival (OS) (A) and cancer‐specific survival (CSS) (B) Kaplan–Meier curves for renal cell carcinoma (RCC) patients treated with laparoscopic nephrectomy stratified by peripheral blood immune (PBIS) scores.

As indicated in Figure S2, low neutrophil levels, high lymphocyte levels, and low monocyte levels were considered to be beneficial indicators of OS and CSS in patients with RCC receiving laparoscopic nephrectomy (all *p* < 0.05). We defined the unfavorable factors as 1 point and the favorable factors as 0 point (Table 2). According to PBIS scoring criteria described above, patients were categorized as PBIS 3 group (*n* = 7), PBIS 2 group (*n* = 69), PBIS 1 group (*n* = 156), and PBIS 0 group (*n* = 206). We found a statistically significant associations between PBIS scores and M‐stage (*p* = 0.008), N‐stage (*p* = 0.006), AJCC stage (*p* < 0.001), and T‐stage (*p* < 0.001) (Table 3). We subsequently used Kaplan–Meier survival curves to assess the prognostic impact of PBIS scores on survival outcomes (Figure 1). It was demonstrated that patients with higher PBIS scores were worse in OS (*p* < 0.001) and CSS (*p* < 0.001) compared to the PBIS 0 group.

**TABLE 2:** PBIS score system^a^.

Score	Variable
0	Neutrophil count ≤4.50
	Lymphocyte count >1.01
	Monocyte count ≤0.46
1	Neutrophil count >4.50
	Lymphocyte count ≤1.01
	Monocyte count >0.46

Abbreviation: PBIS score, peripheral blood immune score.

^a^ High neutrophil count, low lymphocyte count and high monocyte count were defined as a score of 1. Low neutrophil count, high lymphocyte count, and low monocyte count were scored 0. The PBIS score system was obtained by summing the three cell scores for each patient.

**TABLE 3:** Baseline characteristics of the patients according to PBIS score.

Characteristic	PBIS score				*p*‐value
	3	2	1	0	
	*N* = 7	*N* = 69	*N* = 156	*N* = 206	
Age, years					
≤65	6 (85.7)	51 (73.9)	102 (65.4)	157 (76.2)	0.112
>65	1 (14.3)	18 (26.1)	54 (34.6)	49 (23.8)	
Gender					
Male	2 (28.6)	49 (71.0)	110 (70.5)	132 (64.1)	0.077
Female	5 (71.4)	20 (29.0)	46 (29.5)	74 (35.9)	
BMI categorized, kg/m^2^					
<25	4 (57.1)	40 (58.0)	90 (57.7)	113 (54.9)	0.945
≥25	3 (42.9)	29 (42.0)	66 (42.3)	93 (45.1)	
Hypertension					
No	5 (71.4)	38 (55.1)	90 (57.7)	117 (56.8)	0.866
Yes	2 (28.6)	31 (44.9)	66 (42.3)	89 (43.2)	
Diabetes					
No	4 (57.1)	63 (91.3)	129 (82.7)	172 (83.5)	0.081
Yes	3 (42.9)	6 (8.7)	27 (17.3)	34 (16.5)	
Cardiovascular diseases					
No	6 (85.7)	64 (92.8)	133 (85.3)	184 (89.3)	0.394
Yes	1 (14.3)	5 (7.2)	23 (14.7)	22 (10.7)	
Smoking					
No	7 (100.0)	56 (81.2)	129 (82.7)	173 (84.0)	0.627
Yes	0 (0.0)	13 (18.8)	27 (17.3)	33 (16.0)	
T‐stage					
T1	2 (28.6)	43 (62.3)	117 (75.0)	171 (83.0)	<0.001
T2	2 (28.6)	4 (5.8)	10 (6.4)	14 (6.8)	
T3	2 (28.6)	19 (27.5)	26 (16.7)	17 (8.3)	
T4	1 (14.3)	3 (4.3)	3 (1.9)	4 (1.9)	
N‐stage					
N0	5 (71.4)	67 (97.1)	149 (95.5)	200 (97.1)	0.006
N1	2 (28.6)	2 (2.9)	7 (4.5)	6 (2.9)	
M‐stage					
M0	7 (100.0)	61 (88.4)	149 (95.5)	202 (98.1)	0.008
M1	0 (0.0)	8 (11.6)	7 (4.5)	4 (1.9)	
Fuhrman grade					
I	0 (0.0)	7 (9.9)	27 (17.3)	39 (18.9)	0.185
II	5 (71.4)	41 (59.4)	97 (62.2)	130 (63.1)	
III	1 (14.3)	19 (27.5)	28 (17.9)	34 (16.5)	
IV	1 (14.3)	2 (2.9)	4 (2.6)	3 (1.5)	
AJCC stage					
I	2 (28.6)	42 (60.9)	115 (73.7)	167 (81.1)	<0.001
II	2 (28.6)	2 (2.9)	9 (5.8)	13 (6.3)	
III	2 (28.6)	14 (20.3)	24 (15.4)	21 (10.2)	
IV	1 (14.3)	11 (15.9)	8 (5.1)	5 (2.4)	

*Note*: Continuous data are presented as the mean ± standard deviation and categorical data as *n* (%). For categorical variables, *p*‐values were analyzed by chi‐square tests. For continuous variables, the *t*‐test for slope was used in generalized linear models.

Abbreviations: AJCC, American Joint Committee on Cancer; BMI, body mass index; PBIS score, peripheral blood immune score.

### 3.3 Independent prognostic factors for OS and CSS

In addition, univariate and multivariate Cox regression were conducted to find independent risk factors for OS and CSS in patients with RCC who underwent laparoscopic nephrectomy (Tables 4 and 5). The univariate analysis indicated that age, BMI, T‐stage, Fuhrman grade, N‐stage, M‐stage, AJCC stage, and PBIS score (PBIS 1 group: HR = 2.089, 95% CI: 1.089–4.007, *p* = 0.027; PBIS 2 group: HR = 2.578, 95% CI: 1.206–5.509, *p* = 0.015; PBIS 3 group: HR = 13.716, 95% CI: 4.535–41.485, *p* < 0.001) were significantly associated with OS (Table 4). A total of six variables were significant risk factors for CSS in patients with RCC, including T‐stage, Fuhrman grade, N‐stage, M‐stage, AJCC stage, and PBIS score (PBIS 1 group: HR = 2.350, 95% CI: 1.026–5.382, *p* = 0.043; PBIS 2 group: HR = 3.259, 95% CI: 1.293–8.214, *p* = 0.012; PBIS 3 group: HR = 17.362, 95% CI: 4.659–64.695, *p* < 0.001) (Table 5). To evaluate the association of the above parameters with OS and CSS in patients with RCC who underwent laparoscopic nephrectomy, we further performed multivariate Cox regression analysis (Tables 4 and 5). The results revealed that M‐stage, AJCC stage, and PBIS score were still independent prognostic factors for OS (PBIS 1 group: HR = 1.632, 95% CI: 0.844–3.153, *p* = 0.145; PBIS 2 group: HR = 1.239, 95% CI: 0.555–2.766, *p* = 0.601; PBIS 3 group: HR = 16.894, 95% CI: 5.300–53.849, *p* < 0.001) and CSS (PBIS 1 group: HR = 1.800, 95% CI: 0.777–4.168, *p* = 0.170; PBIS 2 group: HR = 1.308, 95% CI: 0.492–3.478, *p* = 0.591; PBIS 3 group: HR = 20.562, 95% CI: 5.048–83.767, *p* < 0.001) in patients with RCC (Tables 4 and 5).

**TABLE 4:** Univariate and multivariate analyses of factors associated with overall survival (OS).

Characteristics	Univariate analyses		Multivariate analyses	
	Hazard ratio (95% CI)	*p*‐value	Hazard ratio (95% CI)	*p*‐value
Age, year				
≤65	Reference		Reference	
>65	1.738 (1.010–2.991)	0.046	‐	0.155
Gender				
Male	Reference			
Female	1.199 (0.690–2.083)	0.520		
BMI categorized, kg/m^2^				
<25	Reference		Reference	
≥25	0.539 (0.301–0.968)	0.038	‐	0.210
Hypertension				
No	Reference			
Yes	0.986 (0.572–1.697)	0.958		
Diabetes				
No	Reference			
Yes	0.668 (0.286–1.561)	0.351		
Cardiovascular diseases				
No	Reference			
Yes	1.094 (0.494–2.421)	0.825		
Smoking				
No	Reference			
Yes	1.125 (0.566–2.235)	0.737		
T‐stage				
T1	Reference		Reference	
T2	1.777 (0.614–5.144)	0.289	‐	0.171
T3	5.958 (3.339–10.633)	<0.001	‐	0.295
T4	4.925 (1.701–14.263)	0.003	‐	0.929
N‐stage				
N0	Reference		Reference	
N1	4.868 (2.296–10.321)	<0.001	‐	0.588
M‐stage				
M0	Reference		Reference	
M1	12.661 (6.939–23.100)	<0.001	6.905 (2.092–22.799)	0.002
Fuhrman grade				
I	Reference		Reference	
II	1.726 (0.665–4.475)	0.262	‐	0.619
III	3.511 (1.284–9.604)	0.014	‐	0.977
IV	17.084 (4.839–60.314)	<0.001	‐	0.053
AJCC stage				
I	Reference		Reference	
II	1.117 (0.261–4.784)	0.881	0.682 (0.151–3.093)	0.620
III	4.970 (2.602–9.492)	<0.001	4.192 (2.159–8.139)	<0.001
IV	11.557 (5.914–22.582)	<0.001	2.773 (0.772–9.960)	0.118
PBIS score				
0	Reference		Reference	
1	2.089 (1.089–4.007)	0.027	1.632 (0.844–3.153)	0.145
2	2.578 (1.206–5.509)	0.015	1.239 (0.555–2.766)	0.601
3	13.716 (4.535–41.485)	<0.001	16.894 (5.300–53.849)	<0.001

Abbreviations: AJCC, American Joint Committee on Cancer; BMI, body mass index; CI, confidence interval; CSS, cancer‐specific survival; OS, overall survival; PBIS, score peripheral blood immune score.

**TABLE 5:** Univariate and multivariate analyses of factors associated with cancer‐specific survival (CSS).

Characteristics	Univariate analyses		Multivariate analyses	
	Hazard ratio (95% CI)	*p*‐value	Hazard ratio (95% CI)	*p*‐value
Age, year				
≤65	Reference			
>65	1.437 (0.728–2.836)	0.296		
Gender				
Male	Reference			
Female	0.886 (0.436–1.802)	0.739		
BMI categorized, kg/m^2^				
<25	Reference			
≥25	0.576 (0.283–1.172)	0.128		
Hypertension				
No	Reference			
Yes	1.650 (0.856–3.181)	0.135		
Diabetes				
No	Reference			
Yes	1.080 (0.449–2.597)	0.863		
Cardiovascular diseases				
No	Reference			
Yes	0.680 (0.208–2.220)	0.523		
Smoking				
No	Reference			
Yes	1.186 (0.519–2.707)	0.686		
T‐stage				
T1	Reference		Reference	
T2	3.269 (1.052–10.158)	0.041	‐	0.002
T3	8.118 (3.833–17.194)	<0.001	‐	0.154
T4	9.375 (3.014–29.157)	<0.001	‐	0.761
N‐stage				
N0	Reference		Reference	
N1	5.733 (2.382–13.799)	<0.001	‐	0.511
M‐stage				
M0	Reference		Reference	
M1	18.675 (9.369–37.224)	<0.001	8.236 (2.086–32.519)	0.003
Fuhrman grade				
I	Reference		Reference	
II	1.728 (0.502–5.946)	0.386	‐	0.283
III	4.985 (1.416–17.546)	0.012	‐	0.684
IV	25.932 (5.579–120.536)	<0.001	‐	0.043
AJCC stage				
I	Reference		Reference	
II	2.153 (0.471–9.841)	0.323	1.253 (0.252–6.227)	0.782
III	6.568 (2.787–15.481)	<0.001	5.376 (2.233–12.944)	<0.001
IV	20.124 (8.815–45.939)	<0.001	4.173 (0.922–18.887)	0.064
PBIS score				
0	Reference		Reference	
1	2.350 (1.026–5.382)	0.043	1.800 (0.777–4.168)	0.170
2	3.259 (1.293–8.214)	0.012	1.308 (0.492–3.478)	0.591
3	17.362 (4.659–64.695)	<0.001	20.562 (5.048–83.767)	<0.001

Abbreviations: AJCC, American Joint Committee on Cancer; BMI, body mass index; CI, confidence interval; CSS, cancer‐specific survival; OS, Overall survival; PBIS score, peripheral blood immune score.

### 3.4 Development and validation of predictive nomograms for predicting OS and CSS of RCC patients

**FIGURE 2:**
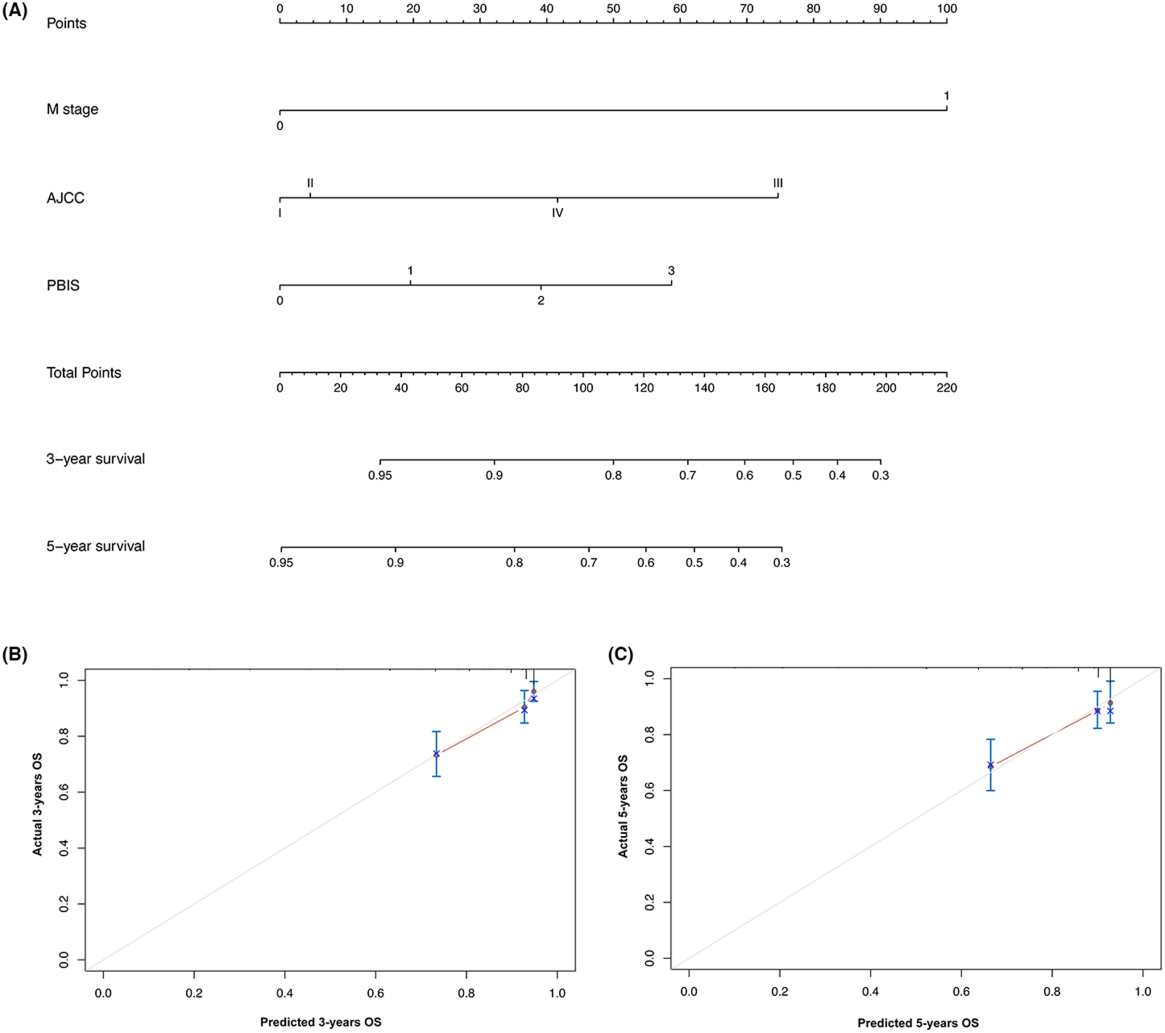
Nomogram for prediction of 3‐year and 5‐year overall survival (OS) in renal cell carcinoma (RCC) patients who underwent laparoscopic nephrectomy (A). 3‐year (B) and 5‐year (C) OS calibration curves for internal validation.

**FIGURE 3:**
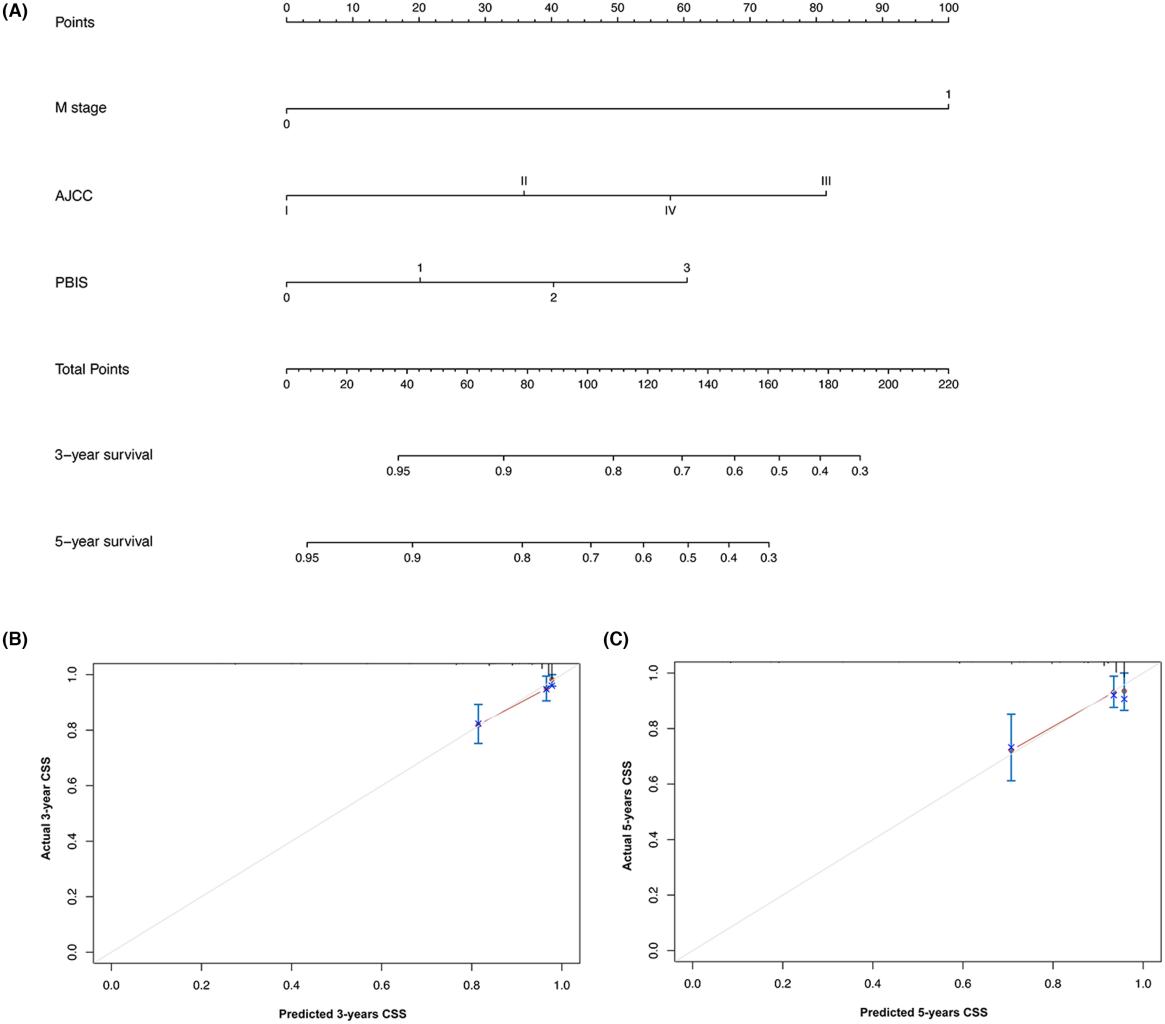
Nomogram for prediction of 3‐year and 5‐year cancer‐specific survival (CSS) in renal cell carcinoma (RCC) patients who underwent laparoscopic nephrectomy (A). 3‐year (B) and 5‐year (C) CSS calibration curves for internal validation.

**FIGURE 4:**
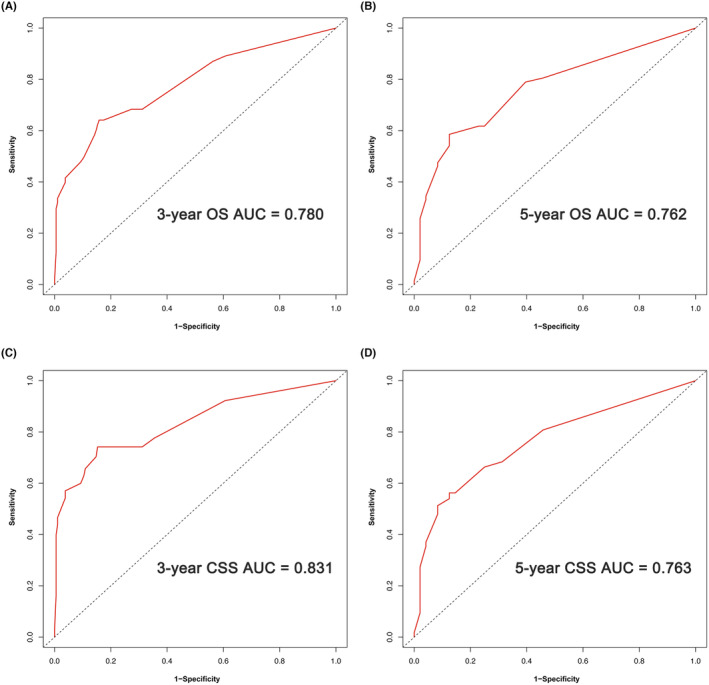
Nomogram prognostic accuracy of receiver operating characteristic (ROC) curve analysis for 3‐year and 5‐year overall survival (OS) (A and B) and 3‐year and 5‐year cancer‐specific survival (CSS) (C and D) in renal cell carcinoma (RCC) patients receiving laparoscopic nephrectomy.

On the basis of the results of multivariate analysis, we created prognostic nomograms to predict 3 year‐ and 5‐year OS (Figure 2A) and CSS (Figure 3A) in patients with RCC receiving laparoscopic nephrectomy. The C‐index of the nomogram integrating PBIS score was 0.770 (95% CI: 0.702–0.838) for OS and 0.828 (95% CI: 0.752–0.904) for CSS to validate the internal performance of the nomogram. By constructing feasibility calibration plots for 3‐year or 5‐year OS (Figure 2B,C) and CSS (Figure 3B,C) in RCC patients receiving laparoscopic nephrectomy, the results indicated the prediction of the nomogram was optimally accorded with the actual observed value. As shown in Figure 4, the 3‐year and 5‐year AUCs of OS for the nomogram were 0.780 and 0.762, respectively, and 3‐year and 5‐year AUCs of CSS for the nomogram were 0.831 and 0.763, respectively, demonstrating that the nomograms had good discrimination ability.

**FIGURE 5:**
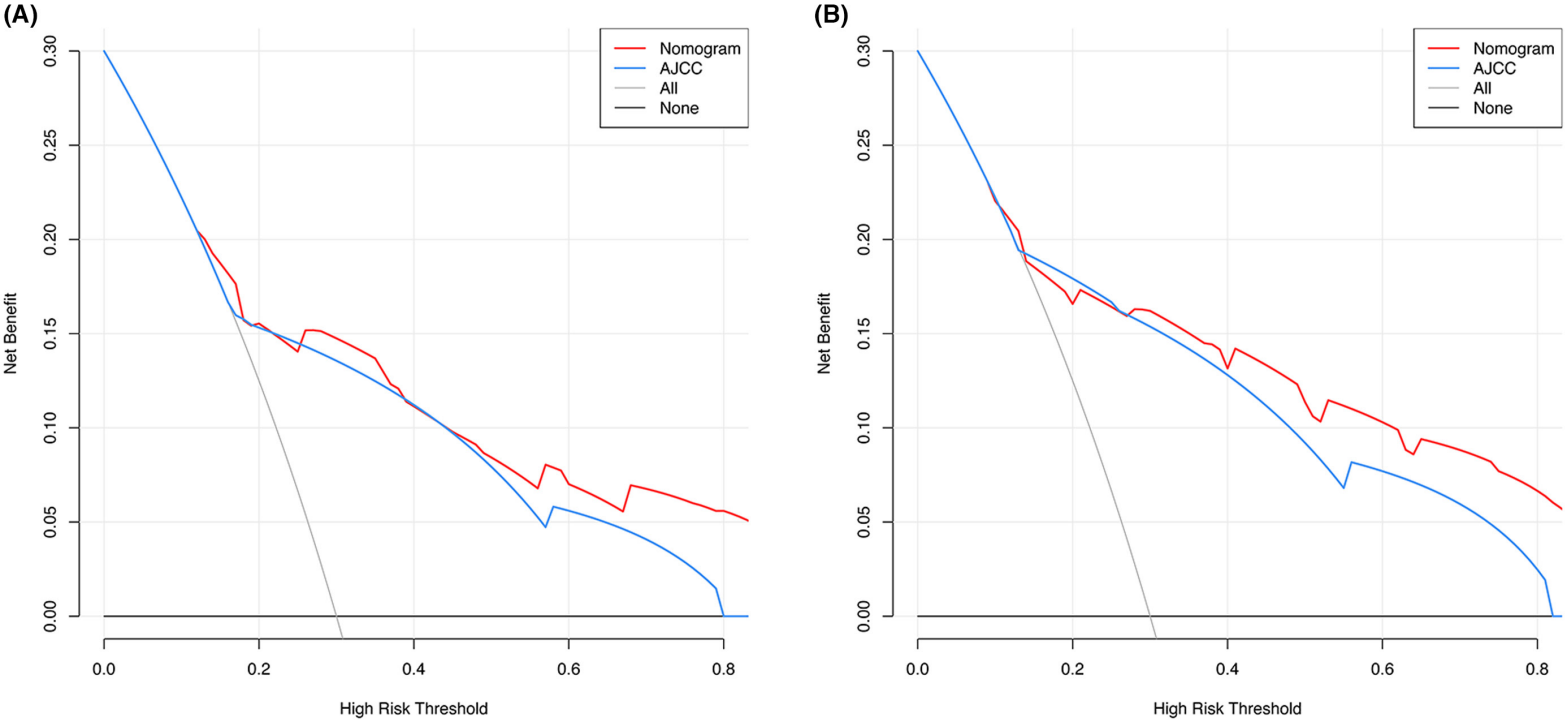
Decision curve analysis (DCA) of nomogram and AJCC stage for OS (A) and CSS (B) survival benefit for renal cell carcinoma (RCC) patients receiving laparoscopic nephrectomy.

**FIGURE 6:**
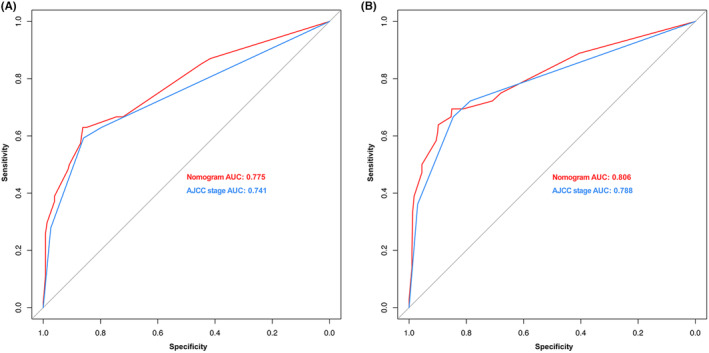
Receiver operating characteristic (ROC) curve analysis of the nomogram and AJCC stage predicting overall survival (OS) (A) and cancer‐specific survival (CSS) (B) in renal cell carcinoma (RCC) patients that underwent laparoscopic nephrectomy.

**FIGURE 7:**
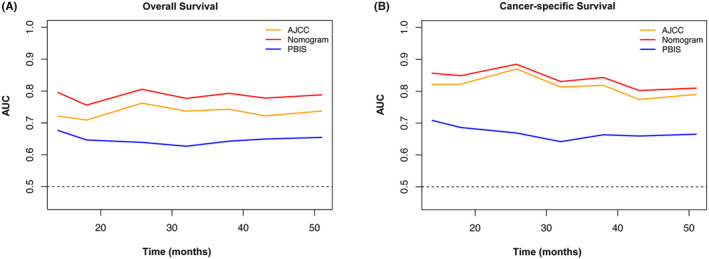
Time‐dependent receiver operating characteristic (ROC) curves of peripheral blood immune (PBIS) scores, nomogram and AJCC stage for overall survival (OS) (A) and cancer‐specific survival (CSS) (B) in renal cell carcinoma (RCC) patients that underwent laparoscopic nephrectomy.

In addition, we compared the clinical benefits of nomograms with those of AJCC staging. The ROC curves and DCA curves were performed to compare the clinical benefits of nomograms with AJCC stage (Figures 5 and 6). Compared to AJCC stage, the DCA curves suggested that nomograms, because it had more net benefit, were better able to predict 3‐year and 5‐year OS (Figure 5A) and CSS (Figure 5B) in patients with RCC. In predicting 3‐year and 5‐year OS and CSS, the AUCs of the nomograms were 0.775 and 0.806, both higher than the AJCC stage of 0.741 and 0.788, respectively, indicating that the predicting prognostic ability of nomograms was more accurate (Figure 6). Finally, the predictive value of nomograms, AJCC stage, and PBIS score were compared by time‐dependent ROC curves. The AUCs of time‐dependent ROC curves revealed that the prognostic capability of nomograms was superior to AJCC stage and PBIS score alone over a wide time period in patients with RCC who underwent laparoscopic nephrectomy (Figure 7).

## 4 DISCUSSION

In the present study, we retrospectively investigated the prognostic significance of preoperative peripheral blood immune cells, including monocytes, neutrophils, and lymphocytes, in RCC patients undergoing laparoscopic nephrectomy. We constructed the PBIS score by considering high neutrophils, low lymphocytes, and high monocytes as favorable prognostic indicators for RCC patients. Multivariate analysis and Kaplan–Meier survival curves revealed that PBIS score was an objective prognostic factor for OS and CSS in RCC patients. In addition, calibration plots, DCA curves and ROC curves showed that nomograms combining PBIS score, M‐stage, and AJCC stage had good predictive ability for OS and CSS. Moreover, compared to AJCC stage and PBIS score, the nomograms were able to predict patient prognosis more accurately. Therefore, the PBIS score, as a novel biomarker, can predict accurately the prognosis of patients with RCC receiving laparoscopic nephrectomy, thus guiding physicians to provide postoperative adjuvant therapy to patients.

Despite some advances in diagnostic and therapeutic approaches for RCC, the overall survival rate for RCC remained low and was accompanied by a high risk of metastasis and recurrence.^14^, ^15^ There was accumulating evidence that the tumor‐related inflammatory and tumor immune microenvironment had an increasingly significant part in the process of occurrence, progression and metastasis of RCC.^16^ Inflammation and immune microenvironment as is the seventh influencing factor in cancer, the immune response of patients to malignant cells is an important factor in the prognosis of malignancy.^17^ As the main circulating granulocytes in humans, neutrophils which reflect the immune and inflammatory status of the host, are biomarkers of various cancers.^18^ Neutrophils promoted tumor cell proliferation and metastatic processes by direct action on tumor cells or by secreting arginase 1, which inhibited the activation of T cells.^19^, ^20^ As shown in previous studies and the results of the present study, high levels of neutrophils were related to adverse prognosis in patients with a range of cancers.^21^, ^22^

During the progression of cancers, monocytes play an essential role in cancer immune evasion, angiogenesis and tumor cell propagation proliferation by differentiating into immunomodulatory cells.^23^ Based on the heterogeneity of monocytes, it can be divided into classical, non‐classical, and intermediate subpopulations on the basis of CD14 and CD16 expression.^24^ Different subsets of monocytes play different effects on tumorigenesis development. Classical monocytes differentiate mainly into M1 macrophages, which play a tumor suppressive and pro‐inflammatory role.^25^, ^26^ By contrast, the remaining two types of monocytes differentiate into M2 macrophages, which contribute to promote tumor‐promoting and immunosuppressive effects.^27^ In addition, the distribution of the subpopulation composition of monocytes is altered in different malignant cancer diseases.^28^ The mutual conversion of different subgroups had been demonstrated to have a promising predictive value for the prognosis of tumor patients.^29^ Lymphocytes, as immune response cells, play a leading role in anti‐tumor effects by generating immune and participating in cytotoxic cell death responses to tumor cells.^30^ Tumor‐infiltrating lymphocytes had an important anti‐tumor immune role in the tumor microenvironment and were associated with better treatment response and outcome.^20^ Previous studies have confirmed that the reduction of lymphocyte count in peripheral blood diminishes the immune response capacity of the body, leading to the emergence of immune tolerance and a decrease in host immune surveillance, and the escape of tumor cells leading to a decrease in the survival rate of tumor patients.^31^ Based on previous studies, and the results we obtained, we found that low levels of monocytes and neutrophils, and high levels of lymphocytes were associated with a positive prognosis for patients with RCC who underwent laparoscopic nephrectomy.

Recently, an increasing number of studies have revealed that many blood inflammatory and immune biomarkers demonstrate promising prognostic factors in RCC. In a retrospective study, Chandrasekaran et al. found that elevated preoperative the ratios of platelet‐to‐lymphocyte ratios (PLR) and neutrophil‐to‐lymphocyte ratios (NLR) were associated with high‐grade and advanced stage RCC.^32^ In a multicenter study, Keiner et al. found that elevated NLR was independently associated with worsening of all‐cause mortality (ACM) and non‐cancer mortality (NCM) in RCC patients, whereas elevated De Ritis could predict cancer‐specific mortality (CSM) in addition to ACM and NCM.^33^ In addition, the novel blood indicator F‐NLR combining preoperative plasma fibrinogen and NLR proved to be a novel and effective prognostic biomarker for RCC patients undergoing laparoscopic nephrectomy.^34^ New combinations based on absolute number of preoperative lymphocyte, monocyte, and neutrophil counts have been constructed and demonstrated to be prognostic indicators for colorectal cancer patients.^35^ Not only that, PBIS had been established as an independent predictor of disease‐free survival (DFS) in patients with locally advanced rectal cancer.^2^ Therefore, in this study, the relationship between PBIS scores and the prognosis of RCC patients was further analyzed. Not only was PBIS score an independent prognostic predictor for RCC patients after laparoscopic nephrectomy, a high PBIS score was also associated with worse 3‐ and 5‐year OS and CSS. In addition, the predictive ability of the nomograms constructed based on PBIS score, M‐stage, and AJCC stage were more effective and accurate for OS and CSS in RCC patients compared with AJCC stage and PBIS score alone. The ability to advise patients on different postoperative treatments for different PBIS score subgroups. As the PBIS 0 group with good immune status, postoperative observation and rehabilitation can be performed to maintain good immune status. The PBIS 3 group, on the other hand, is a fully immunosuppressed state group, which requires close testing of patients and may require the provision of stronger immune supportive therapy. Therefore, because of its excellent prognostic value, the PBIS score can serve as a non‐invasive and reproducible prognostic predictor for patients with RCC who underwent laparoscopic nephrectomy, which may be useful in guiding clinical decisions.

Nevertheless, there were some limitations to the present study. First of all, the fact that this was a retrospective study, where selection bias cannot be ruled out because of the small sample size and the design of the study. In addition, because three hospitals were included in this study, differences in operative technique, medical facilities, and postoperative patient follow‐up between hospitals were difficult to avoid. Third, despite our best efforts to control for potential confounding factors, other comorbidities of the patient or treatment effects due to medication, these may affect the values of preoperative biological indicators. Finally, given the relatively small sample size in this study, more data from other regions, ethnicities, and national medical centers are needed for external validation. In consideration of the above study limitations, we need to conduct a large prospective study to further validate our conclusions.

## 5 CONCLUSION

In conclusion, the PBIS score, a novel prognostic index incorporating preoperative neutrophil, monocyte, and lymphocyte, has been demonstrated to be an effective independent predictor of prognosis in RCC patients treated with laparoscopic nephrectomy. Compared with AJCC stage and PBIS score alone, the nomograms based on a combination of PBIS, M‐stage, and AJCC stage were more accurately and reliably in assessing the prognosis and risk classification of RCC patients, which are promising as a potential biomarker for guiding optimal postoperative treatment decisions for RCC patients.

## AUTHOR CONTRIBUTIONS

**Jinliang Ni:** Conceptualization (equal); data curation (equal); formal analysis (equal); investigation (equal); methodology (equal); project administration (equal); resources (equal); software (equal); supervision (equal); validation (equal); visualization (equal); writing – original draft (lead); writing – review and editing (lead). **Xiaoxiang Yao:** Data curation (equal); formal analysis (equal); methodology (equal); writing – original draft (equal); writing – review and editing (equal). **Wei Song:** Conceptualization (equal); project administration (equal); resources (equal); validation (equal); writing – original draft (equal); writing – review and editing (equal). **Heng Zhang:** Formal analysis (equal); resources (equal); software (equal); writing – original draft (equal); writing – review and editing (equal). **Houliang Zhang:** Methodology (equal); project administration (equal); visualization (equal); writing – original draft (equal); writing – review and editing (equal). **Yidi Wang:** Conceptualization (equal); supervision (equal); writing – original draft (equal); writing – review and editing (equal). **Yifan Zhang:** Project administration (equal); visualization (equal); writing – original draft (equal); writing – review and editing (equal). **Guangchun Wang:** Data curation (equal); funding acquisition (equal); investigation (equal); writing – original draft (equal); writing – review and editing (equal). **Keyi Wang:** Data curation (equal); methodology (equal); project administration (equal); software (equal); writing – original draft (equal); writing – review and editing (equal). **Weipu Mao:** Data curation (equal); formal analysis (equal); methodology (equal); software (equal); writing – original draft (equal); writing – review and editing (equal). **Bo Peng:** Data curation (equal); formal analysis (equal); funding acquisition (equal); resources (equal); supervision (equal); validation (equal); writing – original draft (equal); writing – review and editing (equal).

## FUNDING INFORMATION

This work was funded by National Natural Science Foundation of China (Grant Nos. 81870517; 32070646 and 82270809); Shanghai Association for Science and Technology Commission (Grant Nos. 21142203400, 23JC1401203 and 23JC1401200), Program for Research‐oriented Physician of Shanghai Tenth People's Hospital (Grant No. YNCR2A012 and 2023YJXYSA016), Natural Science Foundation of Jiangsu Province (Grant Nos. BK20230842), Research Personnel Cultivation Programme of Zhongda Hospital Southeast University (Grant Nos. CZXM‐GSP‐RC60) and the National Key Research and Development Program of China (Grant No. 2021YFC2009301).

## CONFLICT OF INTEREST STATEMENT

The authors declare that they have no competing interests.

## ETHICS STATEMENT

The authors are responsible for all aspects of the work and for ensuring that questions relating to the accuracy or completeness of any part of the work are properly investigated and resolved. The study was approved by the Ethics Committee of Zhongda Hospital Southeast University, Shanghai Tenth People's Hospital and Shidong Hospital (SHSY‐IECKY‐4.0/18–68/01 and ZDKYSB077). It was conducted in accordance with the Declaration of Helsinki (as revised in 2013). The patients/participants provided their written informed consent to participate in this study.

## Supporting information


**Figure S1.** Optimal cut‐off values for lymphocyte (A), neutrophil (B), and monocyte (C) were determined by the X‐tile program.

**Figure S2.** Overall survival (OS) and cancer‐specific survival (CSS) Kaplan–Meier curves for renal cell carcinoma (RCC) patients treated with laparoscopic nephrectomy stratified by lymphocyte (A and B), neutrophil (C and D) and monocyte (E and F).

## Data Availability

The datasets generated for this study are available on request to the corresponding author.
