# Supplementary figures and images for: Prognostic value of preoperative combined neutrophil, monocyte, and lymphocyte scores in patients with renal cell carcinoma undergoing laparoscopic nephrectomy: A retrospective study
Source: Cancer Med. 2024 Apr 30;13(9):e7214. doi: 10.1002/cam4.7214 (PMC11058690; doi:10.1002/cam4.7214)

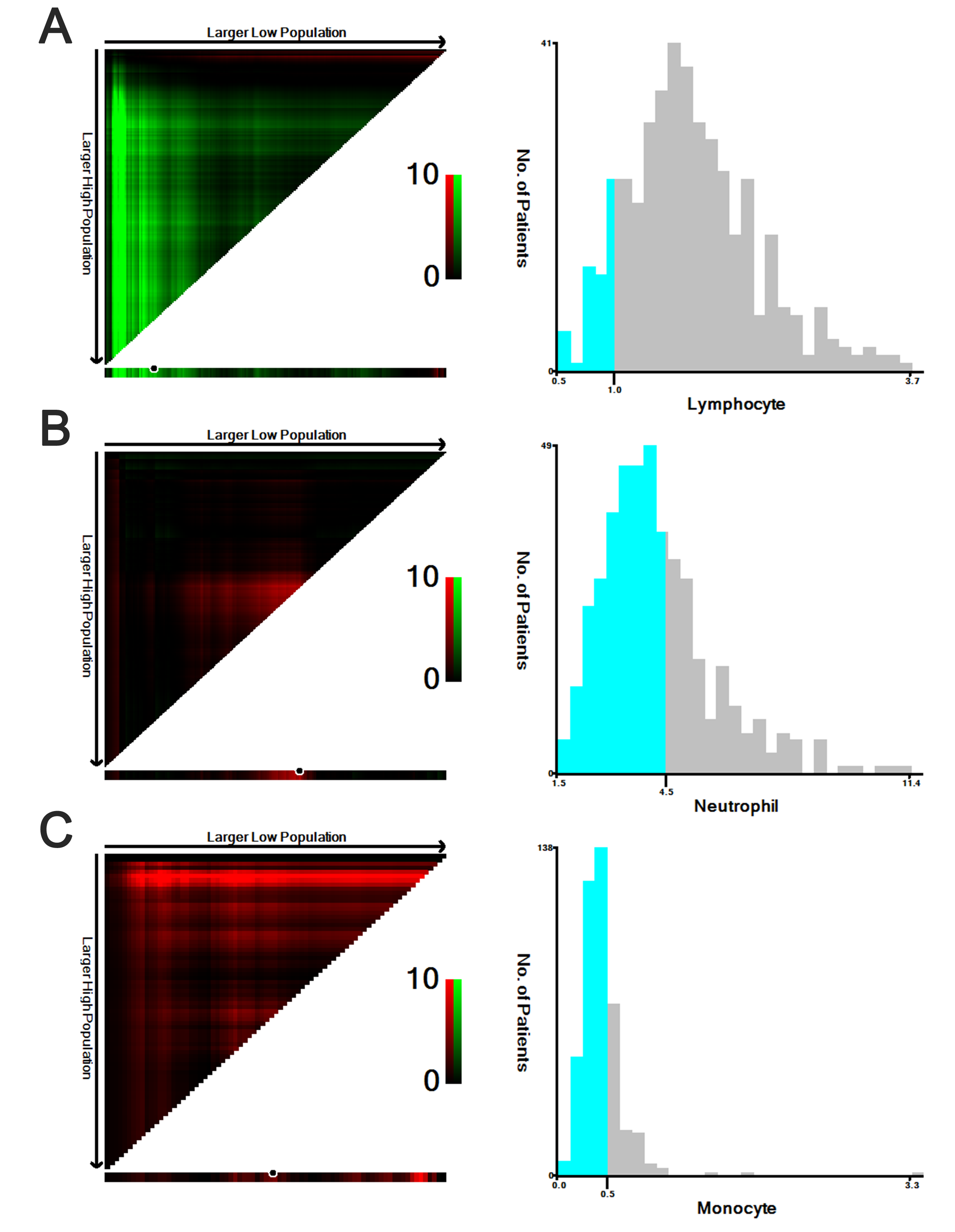

Supplement: Supplementary file 1 — Figure S1. Optimal cut‐off values for lymphocyte (A), neutrophil (B), and monocyte (C) were determined by the X‐tile program. [file CAM4-13-e7214-s001.tif]

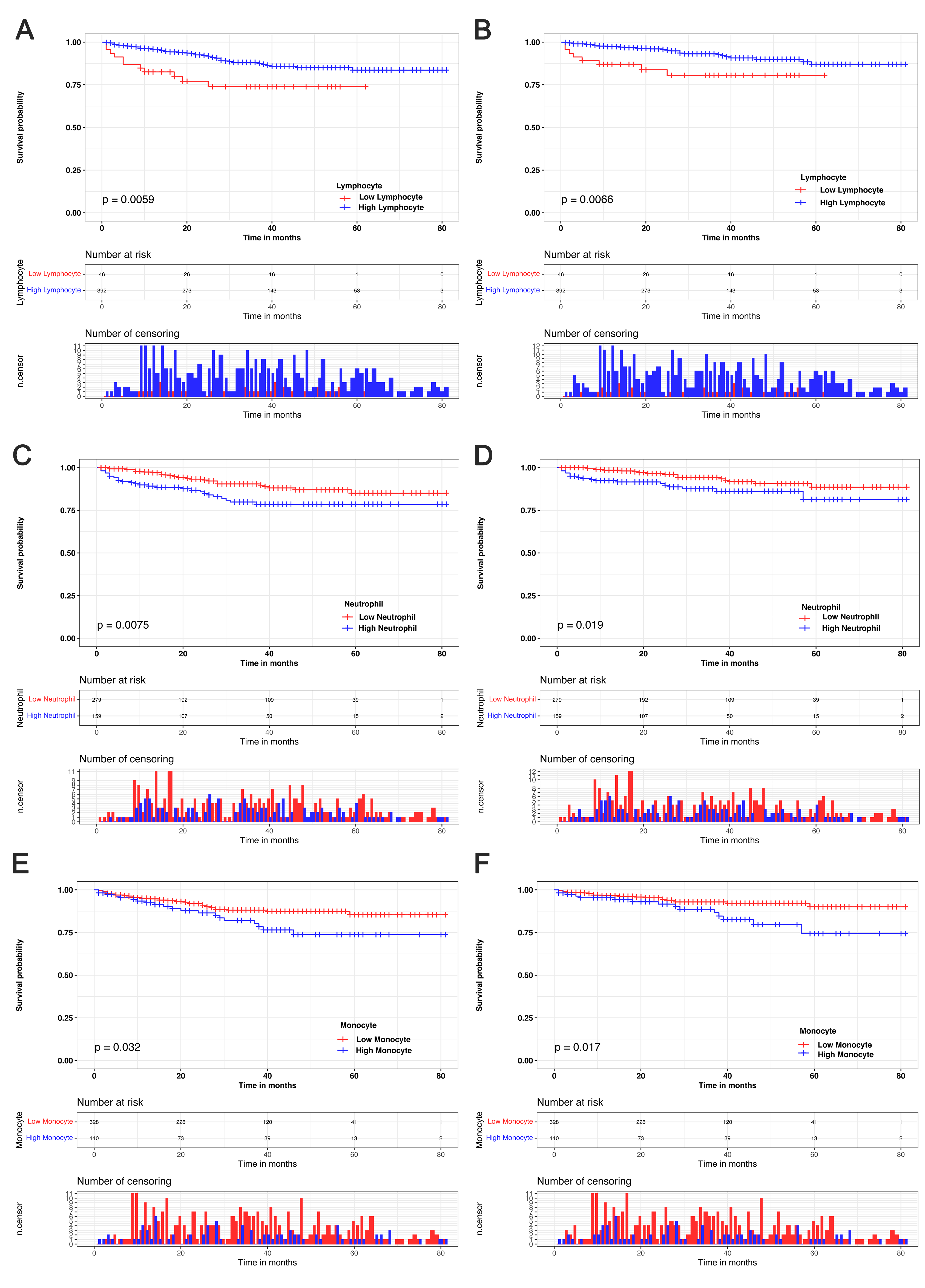

Supplement: Supplementary file 2 — Figure S2. Overall survival (OS) and cancer‐specific survival (CSS) Kaplan–Meier curves for renal cell carcinoma (RCC) patients treated with laparoscopic nephrectomy stratified by lymphocyte (A and B), neutrophil (C and D) and monocyte (E and F). [file CAM4-13-e7214-s002.tif]
